# Giant Hepatic Cyst with Septal Structure: Diagnosis and Management

**DOI:** 10.1155/2013/981975

**Published:** 2013-05-29

**Authors:** Toshihiro Sato, Michitaka Imai, Kazunao Hayashi, Osamu Isokawa, Tatsuya Nomura, Yoshiaki Tsuchiya, Takashi Kawasaki

**Affiliations:** ^1^Department of Gastroenterology, Kashiwazaki General Hospital and Medical Center, 2-11-3 Kitahanda, Kashiwazaki, Niigata 9458535, Japan; ^2^Department of Surgery, Niigata Cancer Center Hospital, Japan; ^3^Department of Pathology, Niigata Cancer Center Hospital, Japan

## Abstract

The hepatic cyst is a common benign liver tumor, and no surgical treatment is necessary. However, it is difficult to correctly diagnose the giant hepatic cyst containing the solid septal structures inside, from the malignant cystadenocarcinomas. The various imaging modalities such as computed tomography, magnetic resonance imaging, and ultrasonography, have been developed and are useful for the diagnosis of these liver tumors. Reviewing the other reports in this paper, the combination of more than 2 modalities will help to diagnose these tumors; however, the malignant potential is unable to be excluded if the tumor is huge. Therefore, the surgical resection should be considered for the huge hepatic cysts with septal structures if the correct diagnosis is unable to be made. For example, when the hemorrhages cause the granulation in the septa which often shows neovascularization, the imaging modalities are unable to define this situation from the malignant tissue with hypervascularity. Therefore, with the careful review of other reports, we conclude that if the imaging studies show the possible malignant potential or the sizing-up is marked, the surgical treatment should be considered with the consent from the patients.

## 1. Introduction

To date, cystic diseases of the liver are being encountered more frequently in the clinical setting because of advancements in various diagnostic imaging modalities. While some cases are easy to diagnose on the basis of medical history and clinical symptoms, such as metastatic hepatic tumors and hepatic abscesses, it becomes difficult to distinguish simple hepatic cysts from malignant diseases in some cases with diverse findings or intracystic hemorrhage and infection [1]. To update the information for the diagnosis of the hepatic cyst and help the therapeutic decision, we reviewed reports showing our representative hepatic cyst case mimicking the biliary cystadenocarcinoma in this paper.

## 2. Clinical Features of Primary Cystic Liver Tumors 

### 2.1. Simple Cyst

Hepatic cysts are the most frequently occurring, benign, space-occupying lesions of the liver. These cysts contain fluid, and their inner walls are covered with a layer of epithelial cells [2]. It can be classified by etiology into congenital and acquired cysts. The former is divided into parenchymal and biliary cysts, and parenchymal cysts can be isolated or polycystic. Acquired cysts are broadly classified as traumatic, inflammatory (including parasitosis), and neoplastic cysts. The ultrasonography (US) shows a well-circumscribed anechoic lesion with increased through-transmission of sound and no evidence of mural nodularity. The computed tomography (CT) shows water-density lesions with sharply defined margins and smooth thin walls. The magnetic resonance imaging (MRI) shows homogeneously hypointense lesion on T1-weighted imaging and homogeneously hyperintense on T2-weighted imaging (Table 1). A majority of benign hepatic cysts are frequency multiple, usually asymptomatic, and only a few centimeters in size, and usually no treatment is required. Treatment becomes necessary, however, when cysts larger than 10 cm in diameter cause pressure symptoms in the surrounding organs, when cysts are accompanied by infection and hemorrhage, or when diagnostic imaging shows evidence of malignancy [3, 4]. 

### 2.2. Complicated Cyst

Complicated cysts are rare occurring, which may be indistinguishable from cystic tumors. Intracystic hemorrhage or infection results in the development of complicated cysts. The cystic hemorrhages, internal honeycomb patterns due to coagulum adhesion to the inner surface, and fibrin deposits that form septa are observed in some simple hepatic cysts on abdominal US. The cystic fluid content can also demonstrate increased echo levels because of an increased plasma component; this hampers differentiation from malignant diseases [1]. Usually, there is no enhancement to coagulum adhesion like a mural nodularity. The same as a simple cyst, the thickening of the cyst wall of a non-bleeding part and an uninfected part is not seen, and the boundary with circumference hepatic tissue is unclear (Table 1). Most of the complicated cysts present clinically with pain and fever, so that the management may need percutaneous drainage or surgical resection [3, 4]. 

### 2.3. Biliary Cystadenocarcinoma

According to the 5th edition of the General Rules for the Clinical and Pathological Study of Primary Liver Cancer, biliary cystadenocarcinoma is defined as a malignant cystic tumor covered by mucus-producing epithelium (similar to bile duct epithelium) that exhibits papillary hyperplasia [5]. It is a rare tumor that accounts for only 0.13% of all primary hepatic cancers [6]. Multilocular lesions are most frequently accompanied by intracystic mucus secretion, and, in women, mesenchymal stromal cells may be present beneath the epithelium. Of late, this tumor has been attracting attention owing to its pathological features [7]. In the 2010 revision of the Classification of Tumors of the Digestive System by the World Health Organization, the term “biliary cystadenocarcinoma/adenoma” was eliminated and the concept of mucinous cystic neoplasm (MCN) was introduced for tumors of the hepatobiliary system. Cystic intraductal papillary neoplasm was cited as a related disease [7–10]. The characteristic appearance is a solitary complex cystic mass with well-defined thick fibrous capsule, internal septations, and mural nodularity. And solid tumor components in a cystic wall as observed on abdominal US and CT suggest a diagnosis of biliary cystadenocarcinoma, and increased contrast uptake on contrast-enhanced CT is a feature that differentiates malignant lesions from simple hepatic cysts [11, 12] (Table 1). 

Biliary cystadenoma is difficult to be differentially diagnosed from cystadenocarcinoma by the imaging studies [13, 14]. 

Reviewing these characteristic features of cystic liver tumors, it is revealed that although the imaging studies have been developed, the careful consideration for the therapeutic decision is necessary since there are so many complicated tumors. Also as there are a few reports showing malignant potential of these tumors, the careful followup is necessary even for the tumors diagnosed as simple hepatic cyst. 

## 3. Huge Cystic Liver Tumor with Septal Structure 

The difficulty of the diagnosis of huge cystic tumor based on the imaging studies is revealed in our representative case which mimicked the cystadenocarcinoma. 

**Figure 1:**
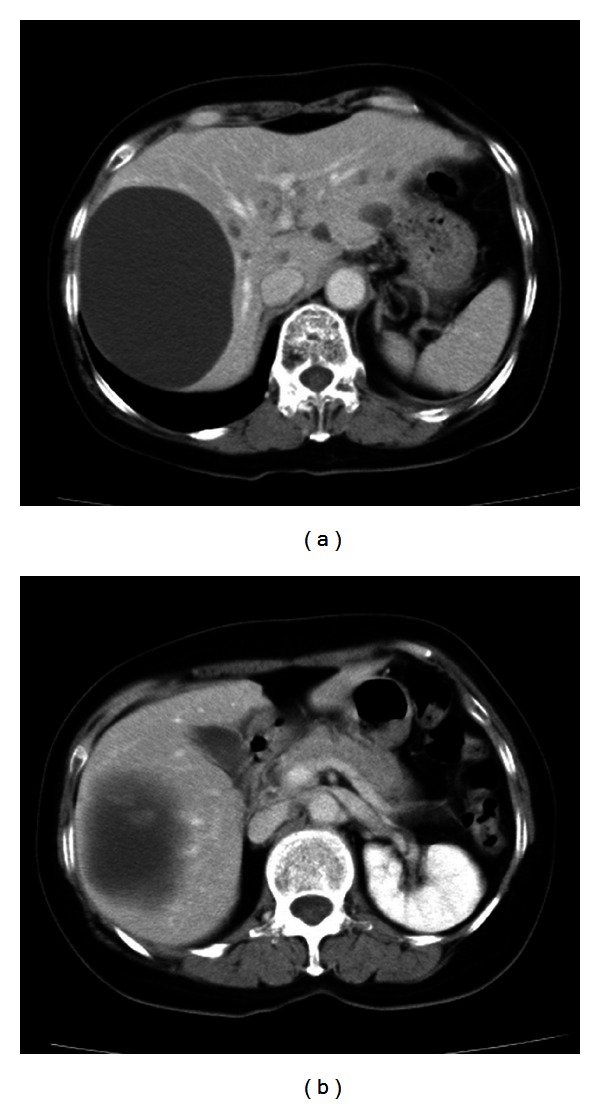
Abdominal contrast-enhanced computed tomography performed in September 2011 shows multiple cysts in both hepatic lobes. Nodular structures can be observed on the caudal side of a giant cyst.

**Figure 2:**
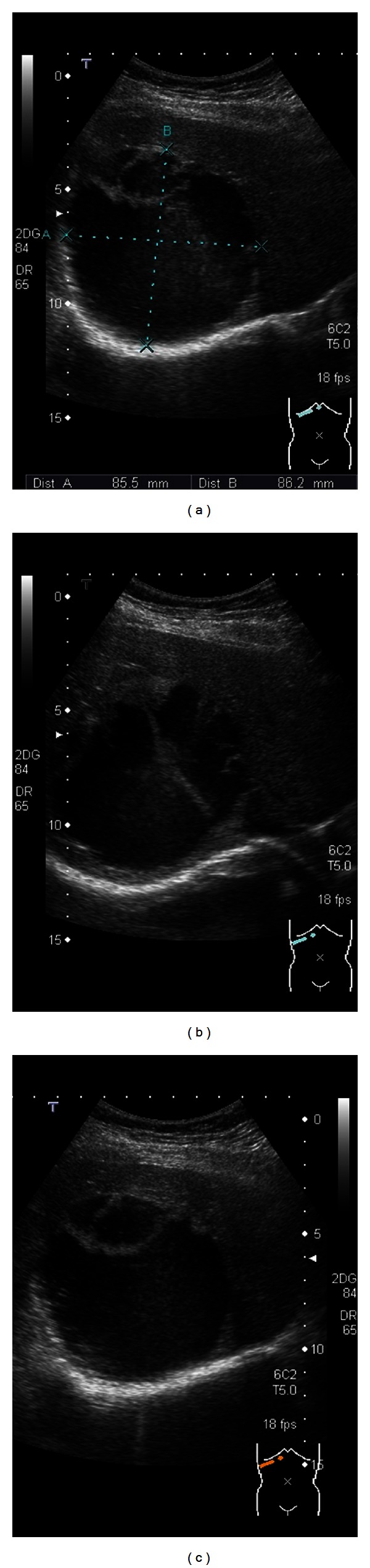
Abdominal ultrasonography performed in January 2012 reveals multiple cysts in both hepatic lobes. The cystic lesion occupying the entire right hepatic lobe shows irregular septal structures and nodules.

**Figure 3:**
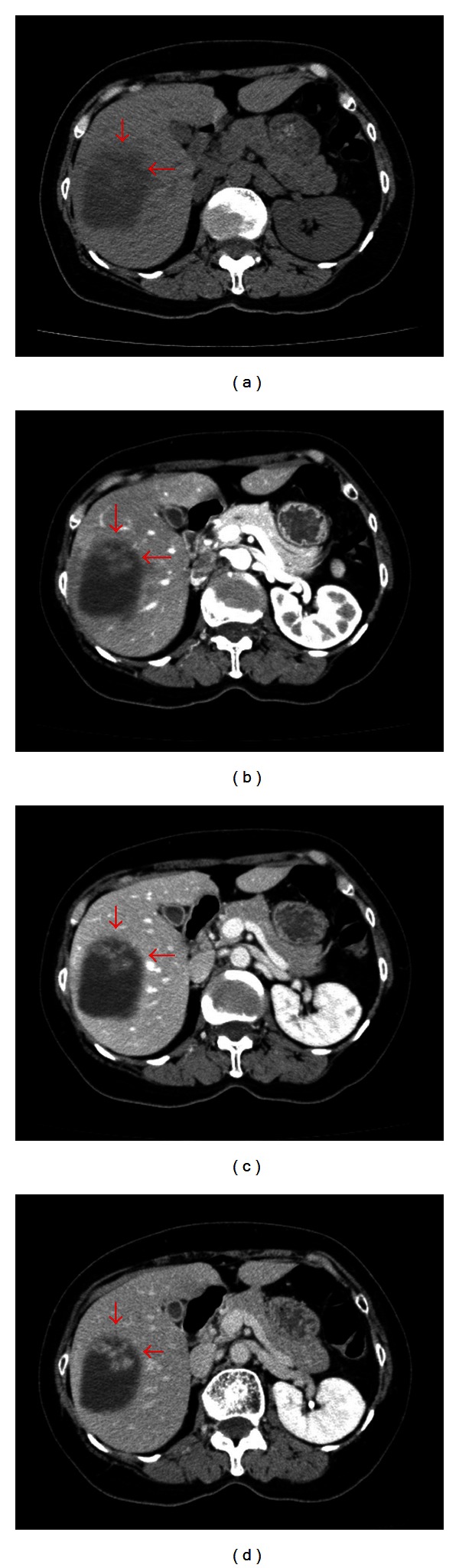
Dynamic contrast-enhanced computed tomography performed in February 2012. (a) Simple, (b) early phase, (c) portal phase, and (d) parallel phase images. The cystic mass occupying the right hepatic lobe was approximately 9.8 × 7.7 × 9.1 cm in size and had slightly increased in size since September 2011. Solid components exhibiting increased contrast uptake are observed at the base and left walls of the cyst (arrows).

**Figure 4:**
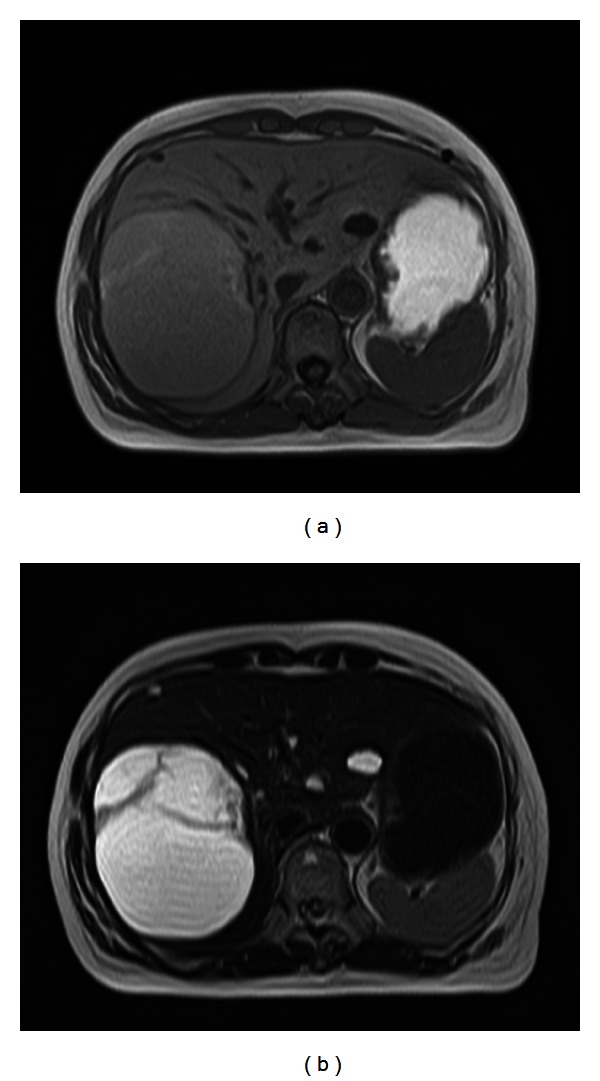
Abdominal magnetic resonance imaging and magnetic resonance cholangiopancreatography performed in February 2012. (a) T1-weighted and (b) T2WI images. The signal intensity within the cystic mass is stronger than that of simple cysts on the T1-weighted image. In addition, multilocular septal structures and nodular solid components are visible at the base. There is no clear communication with the intrahepatic bile ducts.

**Figure 5:**
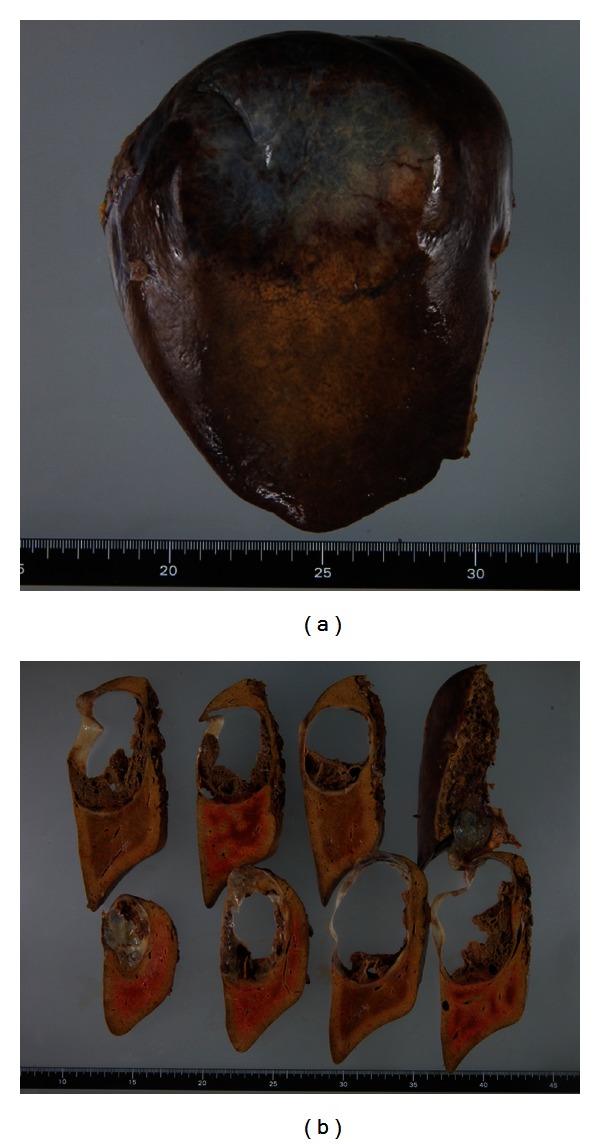
The resected specimen.

**Figure 6:**
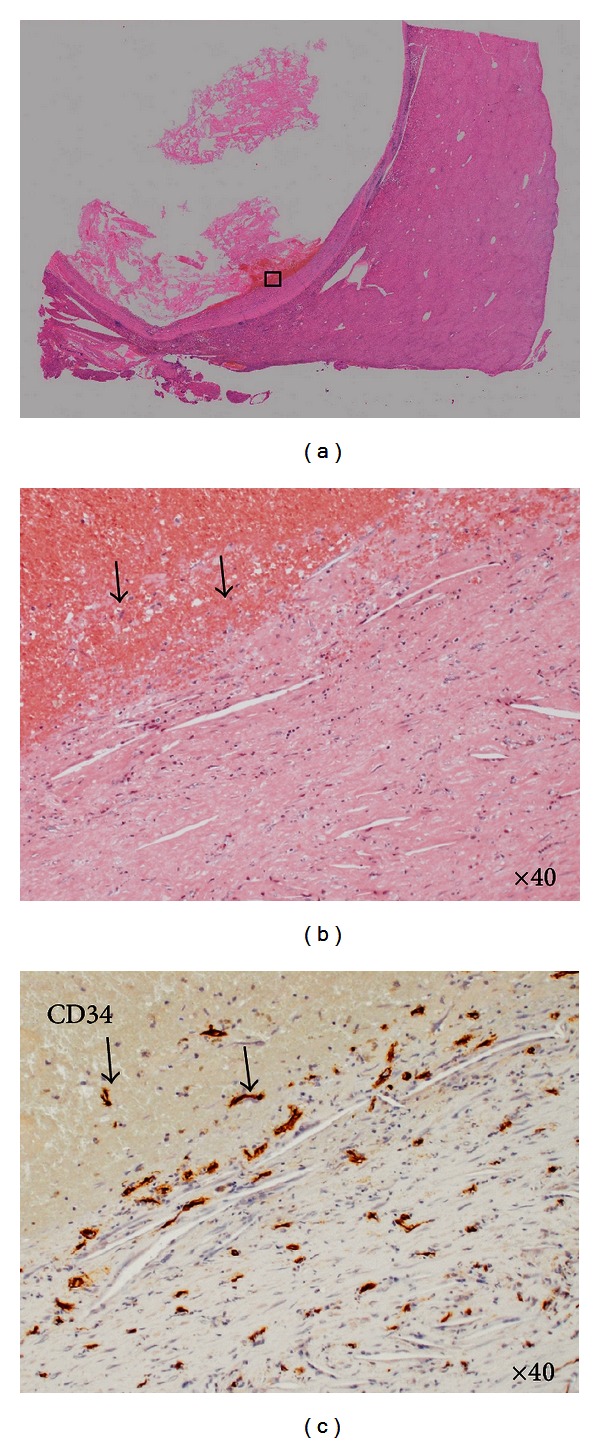
Histopathological images. (a) HE: magnified image, (b) HE: image ×40 magnification, (c) CD34: image ×40 magnification.

Among the multiple cystic lesions in the liver detected by the CT, one of the cysts in the right hepatic lobe was approximately 10 cm in diameter, and nodular structures were seen on the caudal side of a giant hepatic cyst (Figure 1). Abdominal US revealed septal and nodular structures in the giant cyst in the right hepatic lobe (Figure 2). The dynamic CT revealed solid components that exhibited increased contrast uptake at the base and left wall of the giant cyst (Figure 3). Abdominal T1-weighted MRI showed higher signal intensity than that exhibited by simple cysts (Figure 4(a)), and T2-weighted images showed the septal structures (Figure 4(b)). These imaging studies indicate the possibility of the malignant potential of this tumor; however, the resected specimen measured showed no evidence of malignancy. A septum of fibrous connective tissue was observed on the cut surface, and blood serum was present in the cystic fluid (Figure 5). Histopathological examination revealed hemorrhages, fibrin, cholesterol crystals, hemosiderin-laden macrophages, foreign body-type multinucleated giant cells inside the cyst (Figures 6(a) and 6(b)), and neovascularization in the area of granulation tissue (Figure 6(c)) although there were no clear malignant components. Clarification of cells and nuclear vacuoles was observed in some of the hepatocytes in the surrounding hepatic tissue, although no other specific findings were noted. A final diagnosis of a simple hepatic cyst was made. 

## 4. Discussion

The development of various imaging modalities helps the differential diagnosis of cystic liver tumors as summarized in Table 1. However, due to the size and septal structures, some tumors are unable to be correctly diagnosed as a benign simple cyst and the surgical resection is recommended. Since the tumor marker such as CA19-9 increases even in benign tumors [15, 16], it is difficult to make a decision by the serological findings. In these cases as shown in the figures, the puncture of cystic fluid can also be considered, however, due to the numerous of false negative, examination of cyst puncture fluid is hard to be recommended as useful. Moreover, in biliary cystadenocarcinoma, there is possibility of peritoneum sowing by puncture, and even by risking the complications, it is concluded that it is not necessary to perform cyst puncture [15–17]. In recent years, there have been reports that contrast-enhanced US with sonazoid is useful to the diagnosis, because it reveals the clear boundary of a cyst wall and circumference hepatic tissue in early vascular phase and the defect area in Kupffer's phase in biliary cystadenocarcinoma [18]. Furthermore, there have been reports on fluorodeoxyglucose (FDG) accumulation that is consistent with cystadenocarcinoma on FDG-positron emission tomography [19]. And in our case, there was a discrepancy of imaging findings between the modalities as the solid components by CT and the septal structures by MRI. Perhaps, this is a feature which suspects a simple cyst rather than biliary cystadenocarcinoma. It may be the important that the same imaging findings are acquired by two or more modalities. Studies using various diagnostic methods, including novel imaging modalities, are therefore anticipated in the future. However, there are reports on cases of biliary cystadenocarcinoma initially diagnosed as simple hepatic cyst [20] as well as cases of unresectable advanced cancer that undergo long-term followup after an initial diagnosis of cystadenoma [21]. Nevertheless, aggressive surgical resection should be considered when a definitive diagnosis of malignancy cannot be established [16, 22]. 

## 5. Conclusion

Recent reports showed that more than 2 imaging modalities help the correct diagnosis of the hepatic cystic tumors; however, due to the smaller number of the malignant cystic liver tumors and the knowledge about the images, we need to consider the surgical treatment for the cystic tumor if it shows the possible malignant potential.

## Figures and Tables

**Table 1:** Comparison of simple cyst, and complicated cyst and biliary cystadenocarcinoma.

	US	CT	MRI
Simple cyst	Monolocular Anechoic lesion with increased through transmission of sound No structure	Uniformly water-density Smooth thin walls No structure No enhancement	T1WI: homogeneously hypointensity T2WI: homogeneously hyperintensity No structure No enhancement

Complicated cyst	Honeycomb pattern Increased echo levels of cystic fluid	Mural nodularity Various thickness of the walls Unclear boundary Usually no enhancement	T1WI: various intensity T2WI: hyperintense Septal and/or nodular structures Usually no enhancement

Biliary cystadenocarcinoma	Multilocular Septal and/or Nodular structures	Uneven for every locular Thick fibrous capsule Solid tumor component Increased contrast uptake	T1WI, T2WI: various intensity Septal and/or nodular structures Increased contrast uptake

T1WI: T1-weighted imaging,T2WI: T2-weighted imaging.
